# Can a nomogram predict apical prostate cancer pathology upgrade from fusion biopsy to final pathology? A multicenter study

**DOI:** 10.1002/cam4.7341

**Published:** 2024-06-07

**Authors:** Tianrui Feng, Zhen Liang, Yu Xiao, Boju Pan, Yi Zhou, Chengquan Ma, Zhien Zhou, Weigang Yan, Ming Zhu

**Affiliations:** ^1^ Department of Urology Peking Union Medical College Hospital, Peking Union Medical College, Chinese Academy of Medical Sciences Beijing China; ^2^ Department of Pathology Peking Union Medical College Hospital, Peking Union Medical College, Chinese Academy of Medical Sciences Beijing China; ^3^ Department of Urology Tianjin Medical University General Hospital Tianjin China

**Keywords:** magnetic resonance imaging, nomograms, pathology, prostatic neoplasms

## Abstract

**Background:**

This study evaluates the efficacy of a nomogram for predicting the pathology upgrade of apical prostate cancer (PCa).

**Methods:**

A total of 754 eligible patients were diagnosed with apical PCa through combined systematic and magnetic resonance imaging (MRI)‐targeted prostate biopsy followed by radical prostatectomy (RP) were retrospectively identified from two hospitals (training: 754, internal validation: 182, internal–external validation: 148). A nomogram for the identification of apical tumors in high‐risk pathology upgrades through comparing the results of biopsy and RP was established incorporating statistically significant risk factors based on univariable and multivariable logistic regression. The nomogram's performance was assessed via the receiver operating characteristic (ROC) curve, calibration plots, and decision curve analysis (DCA).

**Results:**

Univariable and multivariable analysis identified age, targeted biopsy, number of targeted cores, TNM stage, and the prostate imaging‐reporting and data system score as significant predictors of apical tumor pathological progression. Our nomogram, based on these variables, demonstrated ROC curves for pathology upgrade with values of 0.883 (95% CI, 0.847–0.929), 0.865 (95% CI, 0.790–0.945), and 0.840 (95% CI, 0.742–0.904) for the training, internal validation and internal–external validation cohorts respectively. Calibration curves showed good consistency between the predicted and actual outcomes. The validation groups also showed great generalizability with the calibration curves. DCA results also demonstrated excellent performance for our nomogram with positive benefit across a threshold probability range of 0–0.9 for the training and internal validation group, and 0–0.6 for the internal–external validation group.

**Conclusion:**

The nomogram, integrating clinical, radiological, and pathological data, effectively predicts the risk of pathology upgrade in apical PCa tumors. It holds significant potential to guide clinicians in optimizing the surgical management of these patients.

## 1 INTRODUCTION

Prostate cancer (PCa) remains a leading malignancy among men globally, with approximately 1.41 million new cases diagnosed in 2020, making it the second most common and the fifth deadliest cancer among males worldwide.^1^ Although some tools were made to improve the detection rate of clinically significant prostate cancer (csPCa) and reduce the number of useless biopsies, prostate biopsy is still the golden standard for the diagnosis of PCa.^2^, ^3^ The management of prostate cancer is primarily guided by the Gleason score (GS) obtained from prostate biopsies. While biopsies driven by prostate‐specific antigen (PSA) testing can facilitate early therapeutic intervention for this potentially fatal condition, discrepancies between GS from biopsy samples and final pathology from whole mount sections are prevalent, exhibiting concordance rates between 28% and 68%.^4^ It is important to highlight that approximately 50% of patients initially assessed with a GS of 3 + 3 may undergo an upward revision in their disease status after undergoing radical prostatectomy (RP).^5^ This variability underscores the challenges in accurately staging PCa, potentially leading to suboptimal treatment decisions. Nevertheless, assessing the risk of disease reclassification according to the standard clinical index remains imperfect, resulting in patient anxiety, avoidable treatment, and imprecision in monitoring.^6^, ^7^

The apex of the prostate, pivotal for maintaining continence during RP, has garnered heightened attention owing to its significance in surgical outcomes and the increasing incidence of apical prostate cancer (PCa).^8^, ^9^, ^10^ Optimizing apical dissection to balance cancer control and sphincter preservation is a delicate task that directly impacts postoperative continence.^11^ Previous studies indicated sparing a portion of the apex during RP might potentially improve postoperative continence and potency.^12^ Efforts have been made to avoid unnecessary radical surgery and preserve as much membranous urethral length as possible, which in turn may affect the degree of resection near the prostate apex, thus leading to a higher risk of a positive surgical margin (PSM) of the apical lesion. Although fluorescent confocal microscopy (FCM) was used to assess the urethral and ureteral margins in real‐time and showed adequate diagnostic performance, it has not been widely used in the clinic.^13^ Therefore, the identification of potential clinically significant prostate cancer (csPCa) at the apex is important in terms of surgical approach selection. The nomogram model, serving as a visual tool for multifactorial calibration, has risen to prominence for its capacity to amalgamate various clinical data. This enables personalized risk assessments and aids in the guidance of therapeutic decisions.^14^

This study aims to develop a nomogram that incorporates clinical, radiological, and pathological data to identify patients at high risk of apical tumor pathology upgrades, comparing biopsy results with RP findings to refine surgical approaches and improve patient outcomes.

## 2 MATERIALS AND METHODS

### 2.1 Study design and participants

**FIGURE 1:**
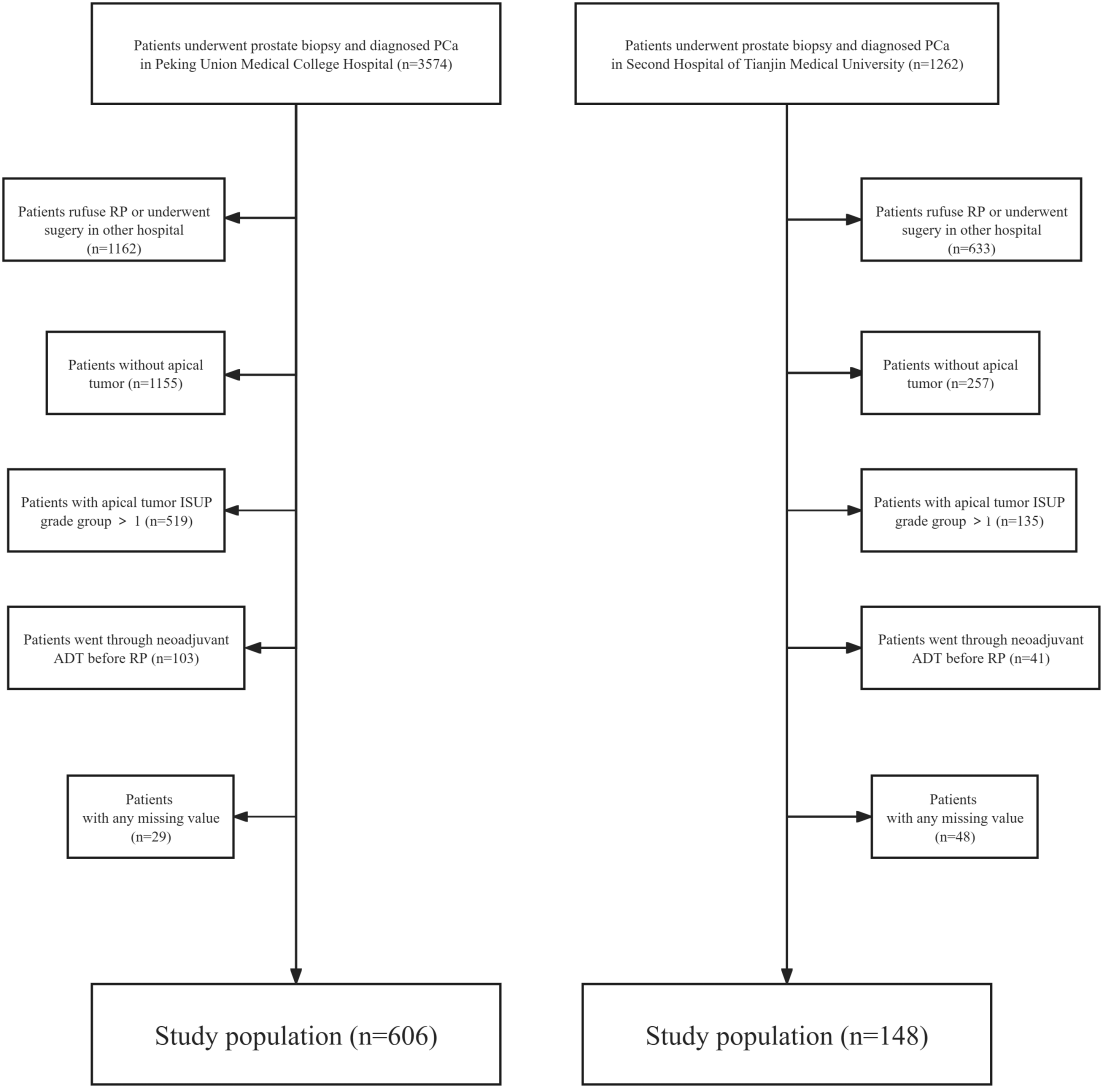
Flow diagram of patients' identification of present study.

This retrospective study was conducted in accordance with Institutional Review Board approved protocols. Personal identifiers were removed and all data were analyzed anonymously. We meticulously selected 606 patients diagnosed with apical low‐risk PCa via cognitive magnetic resonance imaging (MRI) targeted biopsy combined with transrectal ultrasound‐guided (TRUS) transperineal systematic prostate biopsy and then underwent RP at Peking Union Medical College Hospital (PUMCH) between January 2018 and October 2022. A total of 148 patients were recruited in Tianjin Medical University General Hospital (TMUGH) from September 2018 to September 2021, adhering to the following inclusion criteria. Biopsy indications included: (1) total serum PSA > 10.0 ng/mL; (2) total serum PSA between 4.0 and 10.0 ng/mL with a free to total PSA ratio (f/t PSA) <0.16 or a PSA density (PSAD) >0.15; (3) abnormal digital rectal examination (DRE); and (4) suspicious findings on TRUS or mpMRI. Apical tumors are defined as malignant lesions located exclusively within the apical region of the prostate, without consideration of malignancies in other locations. As a result, patients with PCa confined only to nonapical zones are not included in this study. According to the National Comprehensive Cancer Network (NCCN) Guidelines, a low‐risk PCa lesion detected on a prostate biopsy is classified as Grade Group 1.^15^ A stringent set of exclusion criteria was developed to determine the eligibility of patients for participation at both research institutions. These criteria include: (1) Patients without PCa according to biopsy results. (2) Enrollment was limited to patients with complete baseline clinicopathological information and follow‐up data. Any case lacking in this respect was excluded from consideration. (3) Patients who underwent neoadjuvant androgen deprivation therapy before undergoing RP were excluded. Written informed consent was obtained from all enrolled patients. The patient selection flow chart is demonstrated in Figure 1. The study was carried out in accordance with the Declaration of Helsinki and was approved by the Research Ethics Committee of Peking Union Medical College (I‐22PJ417).

### 2.2 MpMRI protocol and scoring criteria

In our center, all patients scheduled for a prostate biopsy undergo a prebiopsy multiparametric MRI (mpMRI) examination. This mpMRI is performed within 1 month following the initial diagnosis of elevated PSA levels in our outpatient department. All MRI images were acquired with a 3‐mm section thickness. T2‐weighted images in the sagittal, coronal, and axial planes, diffusion‐weighted images (b value up to 1500 s/mm^2^) in the axial plane, dynamic contrast‐enhanced images were all obtained based on the European Consensus Meeting (ESUR) on standardization of prostate MRI (More details in Table S1)^16^ Prostate imaging‐reporting and data system (PI‐RADS) score of 3 or greater subsequently underwent a prostate biopsy. Two experienced radiologists with at least 5 years of experience and sub‐specialization in genitourinary interpreted the mpMRI images and marked the regions of interest. Inter‐reader agreement for PI‐RADS was evaluated with Cohen's kappa. As conventionally classified, *κ* values of 0–0.20 defined poor agreement; 0.21–0.40, fair agreement; 0.41–0.60, moderate agreement; 0.61–0.80, substantial agreement; and 0.81–1.0, nearly perfect agreement.^17^

### 2.3 Details of biopsy, surgery, and specimen pathology

With the patient in the lithotomy position, a standard systematic prostate biopsy was initially conducted, two cores based on the prostate biopsy protocol were performed at the apical site, and then 2–5 targeted samples from each MRI region of interest were obtained.^18^ The horizontal section of the prostate was divided into 12 (12‐core biopsy) areas numbered as shown in Figure S1. Finally, each core specimen was stored, labeled, fixed in 10% formaldehyde solution, and sent for pathological examination. The 2014 ISUP system was applied to assess the GS. Laparoscopy RP was conducted through an extraperitoneal approach and five trocar techniques.^19^ Considering the low amount of lymph node metastasis found in the low intermediate‐risk group, it is arguable that pelvic lymphadenectomy may be omitted for prognostic reason according to the standard of NCCN. All biopsy and whole‐mount surgical pathology results were reviewed and reported by a single fellowship‐trained genitourinary surgical pathologist with at least 15 years of experience. The apical tumor in the biopsy specimen was identified as prostate cancer present in core number 11, number 12, or both. The RP specimens were systematically sectioned and entirely embedded into 5 mm thick blocks. We defined an apical tumor as either the complete tumor or a portion thereof that is situated within 1 cm of the distal end of the RP specimen. Oncological outcomes for apical lesions, as determined from both biopsy and RP specimens, were evaluated in accordance with the TNM 2002 classification system. These outcomes were then directly compared to examine their clinical relevance and consistency.

### 2.4 Outcome measures and data analysis

All 754 patients from the two hospitals were utilized as a single training cohort for the development of the model and subsequent internal validation. Of these, 606 patients from PUMCH were randomly allocated into two groups in a 7:3 ratio, yielding subsets of 424 and 182 patients through the use of random numbers. The latter group, consisting of 182 patients, was chosen for internal validation. Additionally, 148 patients from TMUGH were enlisted as an internal–external validation cohort. The training cohort was applied for the following univariable, multivariable analysis, and model building, while the validation cohort was used to validate the feasibility of the proposed model. Clinical data were collected from hospital electronic medical records, including age, PSA, free to total PSA (f/t PSA), prostate volume (PV), PSAD, DRE findings, PI‐RADS score, number of cores taken, number of cores targeted, number of positive cores, TNM stage, biopsy GS, and International Society of Urological Pathology (ISUP) histopathological findings. PV was calculated from mpMRI measurements of the prostate using the ellipsoid formula (height × width × length × π/6).

The normality of data distribution was first assessed using the Shapiro–Wilk test. The mean ± SD was applied to describe data in a normal distribution, while the median and interquartile range (IQR) were applied for data in a skewed distribution. The univariable logistic regression analysis was initially applied to assess different variables and calculate the odds ratio (OR) with a 95% confidence interval (95% CI). Then, potential statistical significance (*p* < 0.05) variables were selected for a multivariable logistic analysis by performing both‐direction stepwise selection to confirm the final variables. A nomogram for the identification of apical tumors in high‐risk pathology upgrades through comparing the results of biopsy and RP was established incorporating statistically significant results based on multivariable logistic regression. The area under the curve (AUC) was generated to investigate the discriminative ability of our nomogram. The calibration curves were then generated to compare the nomogram‐predicted probabilities with actual observed outcomes, establishing the model's accuracy. Additionally, the decision curve analysis (DCA) quantified the nomogram's clinical utility by measuring net benefits across various threshold probabilities in both training and validation cohorts, with its clinical significance inferred from the model's deviation from the “All” and “None” reference curves.^20^ The analysis was performed using “R” for Windows version 4.01 by the rms package.

Biochemical recurrence (BCR) analysis was generated from the initial time of RP until BCR or the last available follow‐up. Subsequent follow‐up was assigned every 3 months for 2 years, then every 6 months for 3 years, and yearly afterward for each patient. BCR post‐RP is defined as at least two consecutive PSA values that are 0.2 ng/mL or higher^21^ and assessed through the Kaplan–Meier analysis. The relationship between apical tumor pathology upgrade and positive surgical margin was examined using the Chi‐square test, with a significance level set at *p* < 0.05 (two‐sided).

## 3 RESULTS

### 3.1 Patient characteristics and mpMRI results

**FIGURE 2:**
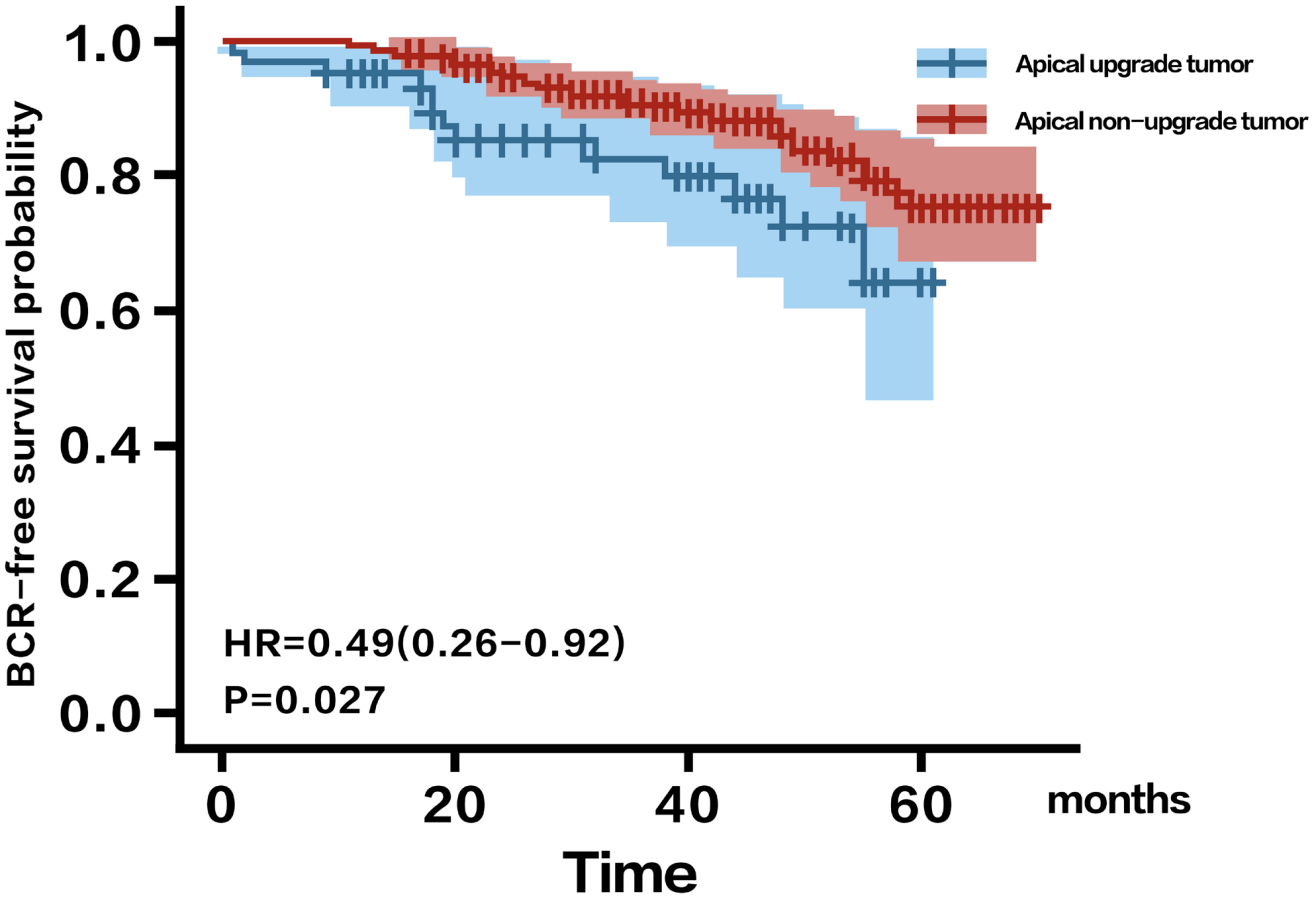
Kaplan–Meier curve showed the apical tumor pathology upgrade group had a significantly higher BCR rate than the non‐upgrade group.

A total of 754 patients with apical PCa who met the inclusion criteria were retrospectively selected from two centers. The median age of the included patients was 65 (IQR: 61–70) years. Key baseline parameters, including PSA, PSAD, and f/t PSA, were recorded with medians of 11.32 ng/mL (IQR: 7.17–19.01), 0.307 ng/mL/mL (IQR: 0.188–0.571), and 0.107 (IQR: 0.078–0.139), respectively. Table 1 shows the clinicopathologic features of these included patients and no clinical significance was detected between the training cohort and validation cohort (Table 2). According to our results, the nonupgrade group had a significantly lower BCR rate than the apical tumor pathology upgrade group. (HR 0.40, 95% CI 0.26–0.92, *p* < 0.001; Figure 2) with *p* < 0.05. A total of 230 patients were diagnosed with PSM. (upgrade group: 100; nonupgrade group: 130) and according to our results of the chi‐square test, patients with apical tumor pathology upgrade were more likely to suffer from PSM (*p* = 0.024). There was fair to moderate agreement for pairwise combination of readers with lesions PI‐RADS ≥3 with *κ* coefficients of 0.38 (95% CI 0.18–0.58), 0.31 (95% CI 0.09–0.53), and 0.54 (95% CI 0.35–0.72), and moderate agreement for pairwise combinations of readers with lesions PI‐RADS ≥4 with *κ* coefficients of 0.50 (95% CI 0.32–0.67), 0.49 (95% CI 0.31–0.68), and 0.54 (95% CI 0.37–0.71).

**TABLE 1:** Baseline characteristics of included patients.

	Upgrade (*n* = 271)	Nonupgrade (*n* = 483)	*p*
Age (median, IQR)	67 (63–72)	64 (60–68)	<0.001
PSA (median, IQR)	12.47 (7.42–19.96)	9.64 (6.38–19.74)	0.021
PSAD (median, IQR)	0.325 (0.283–0.532)	0.292 (0.183–0.587)	0.032
F/t PSA (median, IQR)	0.091 (0.077–0.145)	0.123 (0.089–0.176)	0.043
PV (median, IQR)	44 (34–54)	35 (28–45)	0.006
Gleason score on RP specimens			
3 + 3 (*n*, %)	0 (0)	90 (18.6)	<0.001
3 + 4 (*n*, %)	84 (30.9)	168 (34.8)	0.164
4 + 3 (*n*, %)	78 (28.7)	108 (22.3)	0.053
>7 (*n*, %)	109 (40.2)	117 (24.2)	<0.001
TNM			
T2a (*n*, %)	101 (37.2)	112 (23.2)	0.001
T2b (*n*, %)	38 (14.0)	68 (14.1)	0.533
T2c (*n*, %)	132 (48.7)	303 (62.7)	<0.001
PI‐RADS			
3 (*n*, %)	30 (11.1)	74 (15.3)	0.062
4 (*n*, %)	78 (28.7)	228 (47.2)	<0.001
5 (*n*, %)	163 (60.1)	181 (37.4)	<0.001
Recurrence (*n*, %)	29 (10.7)	37 (7.6)	0.018
Postive margin (*n*, %)	100 (36.9)	130 (26.9)	0.003

Abbreviations: F/t PSA, free to total PSA; IQR, interquartile range; PI‐RADS, prostate imaging‐reporting and data system; PSA, prostate‐specific antigen; PSAD, PSA density; PV, prostate volume; RP, radical prostatectomy.

**TABLE 2:** Baseline characteristics of training group and validation group.

	Training group (*n* = 754)	Internal Validation group (*n* = 182)	Internal–External Validation group (*n* = 148)	*p* Value
Age (median, IQR)	67 (62–70)	67 (62–69)	69 (64–68)	0.698
PSA (median IQR)	12.32 (7.33–19.39)	11.02 (6.87–19.93)	14.45 (7.79–19.47)	0.929
PSAD (median, IQR)	0.369 (0.224–0.517)	0.285 (0.262–0.573)	0.375 (0.288–0.518)	0.385
F/t PSA (median, IQR)	0.116 (0.082–0.151)	0.109 (0.071–0.142)	0.124 (0.086–0.158)	0.812
PV (median, IQR)	34 (21–41)	35.0 (26–44)	39.0 (31–49)	0.560

Abbreviations: F/t PSA, free to total PSA; IQR, interquartile range; PSA, prostate‐specific antigen; PSAD, PSA density; PV, prostate volume.

### 3.2 Univariable and multivariable analysis for apical tumor pathology upgrade

**FIGURE 3:**
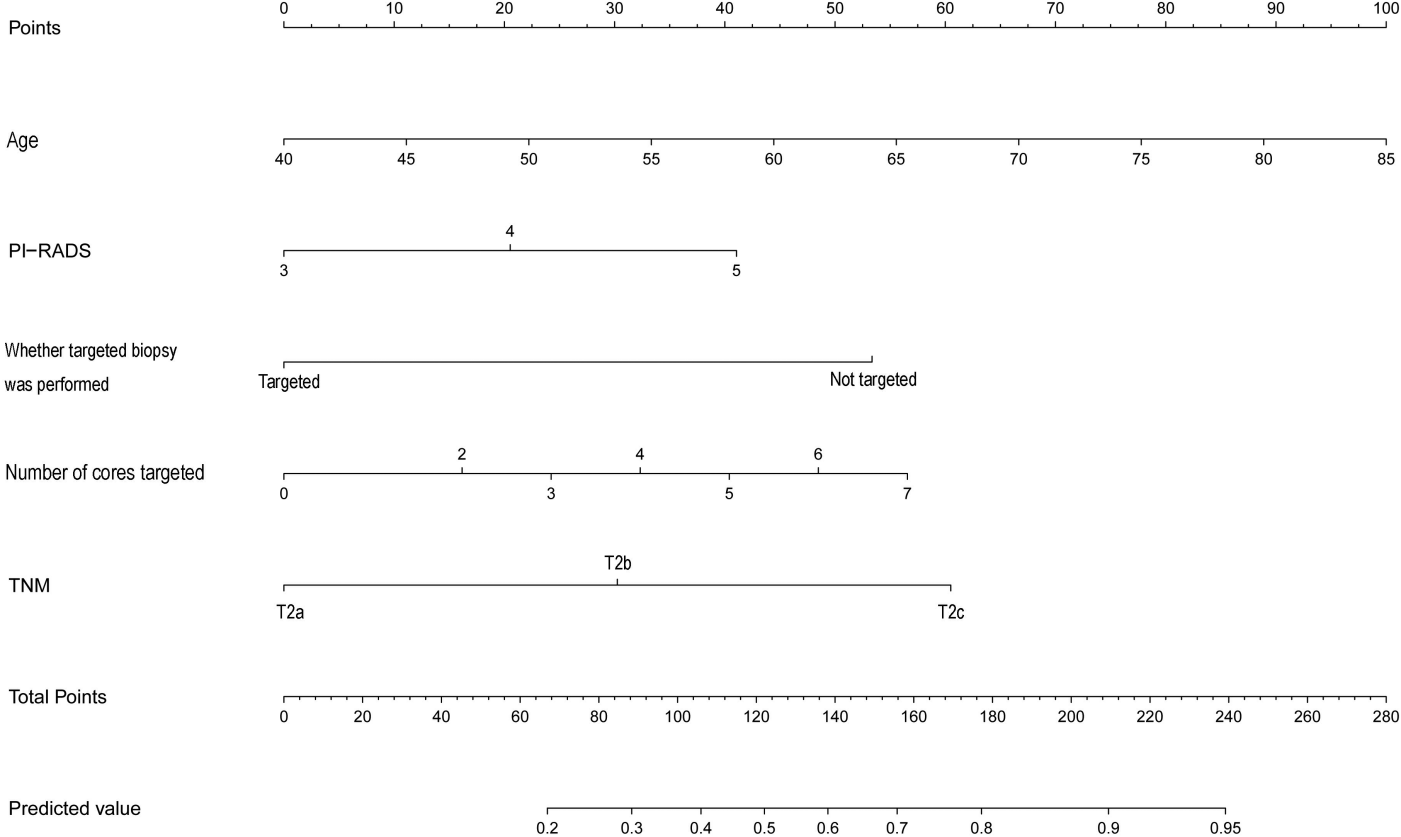
Nomogram predicting apical tumor pathology upgrade.

**FIGURE 4:**
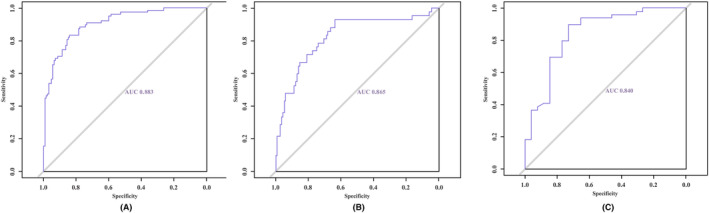
(A) Receiver operating characteristic (ROC) curve of the nomogram for predicting apical tumor pathology upgrade in training cohort (B) ROC curve of the nomogram for predicting apical tumor pathology upgrade in internal validation cohort (C) ROC curve of the nomogram for predicting apical tumor pathology upgrade in internal–external validation cohort.

The statistically significant different variables between nonupgraded and upgraded groups for the apical zone were age, PSAD, target biopsy, number of cores taken, number of cores targeted, number of positive cores, TNM stage, PI‐RADS score (*p* < 0.05). A multivariable analysis revealed that age (OR = 1.075 [95% CI: 1.025–1.127], *p* < 0.001), target biopsy (OR = 0.113 [95% CI: 0.060–0.215], *p* < 0.001), number of cores targeted (OR = 0.693 [95% CI: 0.580–0.829], *p* < 0.001), TNM stage (OR = 6.274 [95% CI: 1.815–21.692], *p* = 0.004), and PI‐RADS score (OR = 5.602 [95% CI: 3.303–9.501], *p* < 0.001) were significant predictors of pathology upgrade (Table 3). Figure 3 shows the nomogram constructed for the biopsy GS upgrade based on the coefficient of the aforementioned six significant predictors. The AUCs were 0.883 (95% CI, 0.847–0.929), 0.865 (95% CI, 0.790–0.945), and 0.840 (95% CI, 0.742–0.904) in the training, internal validation and internal–external validation datasets, respectively (*p* = 0.169), which indicated that the model had a good discrimination ability (Figure 4A–C).

**TABLE 3:** Multivariable analysis for apical tumor pathology upgrade.

	Apical tumor upgrade			
			95% CI for odds ratio	
Characteristics	*p*‐Value	Odds ratio	Lower	Upper
Age	<0.001	1.075	1.025	1.127
PSA	0.931	1.000	0.990	1.011
F/t PSA	0.832	0.840	0.373	1.893
PV	0.325	0.989	0.970	1.009
PSAD	0.039	2.426	1.066	6.097
DRE	0.820	0.932	0.511	1.701
TNM stage	0.004	6.274	1.815	21.692
PI‐RADS	<0.001	5.602	3.303	9.501
Target biopsy	<0.001	0.113	0.060	0.215
Total biopsy core number	<0.001	0.875	0.817	0.938
Number of cores targeted	<0.001	0.693	0.580	0.829
Number of positive cores	<0.001	1.443	1.287	1.618

Abbreviations: CI, confidence interval; DRE, digital rectal examination; F/t PSA, free to total PSA; IQR, interquartile range; PI‐RADS, prostate imaging‐reporting and data system; PSA, prostate‐specific antigen; PSAD, PSA density; PV, prostate volume; RP, radical prostatectomy.

**FIGURE 5:**
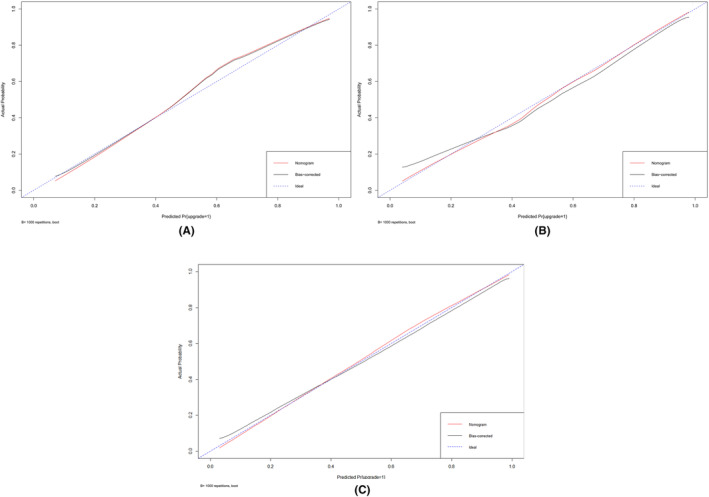
(A) Calibration curve of the nomogram for predicting apical tumor pathology upgrade in training cohort (B) Calibration curve of the nomogram for predicting apical tumor pathology upgrade in internal validation cohort (C) Calibration curve of the nomogram for predicting apical tumor pathology upgrade in internal–external validation cohort.

**FIGURE 6:**
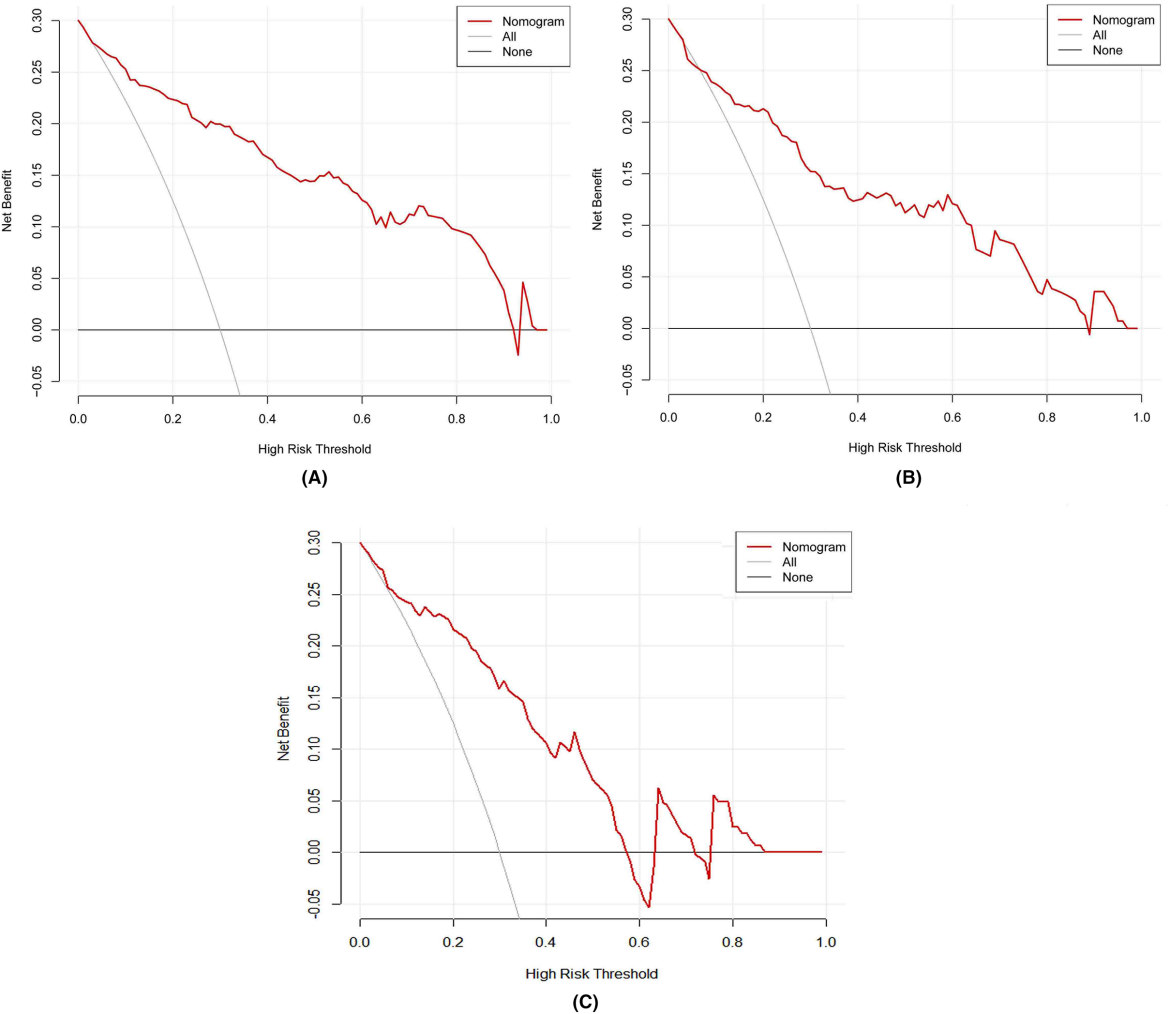
(A) Decision curve analysis (DCA) of the nomogram for predicting apical tumor pathology upgrade in training cohort (B) DCA of the nomogram for predicting apical tumor pathology upgrade in internal validation cohort (C) DCA of the nomogram for predicting apical tumor pathology upgrade in internal–external validation cohort.

Moreover, the calibration curve of the nomogram for the probability of apical tumor pathology upgrade demonstrated a good agreement between prediction and observation for both sets, the slope of the calibration curve was close to the 45‐degree line (intercept = 0.014, 0.067, and 0.129), respectively, (Figure 5A–C) which means the model's predicted probabilities are largely consistent with the observed outcomes. DCA was used to test the clinical usefulness of the model. DCA curves in the training and validation cohorts also demonstrated robust potential for clinical application and indicated significant net benefits across the range of 0–0.9 for the training and internal validation group, and 0–0.6 for the internal–external validation group. (Figure 6A–C). In this study, all participating patients were subjected to RP with comprehensive apical dissection. However, our nomogram reveals that such an extensive procedure could have been avoided for 196 of these patients. According to the nomogram's predictions, the likelihood of an apical tumor pathology upgrade in these cases was below 20%.

## 4 DISCUSSION

In the present study, we established a nomogram capable of accurately predicting the risk of pathology upgrades in PCa. So far as we know, this is the first study investigating the prediction of apical tumor GS upgrade through the comparison of biopsy results and postoperative pathology, accompanied by the follow‐up analysis to explore the prognosis and pathological features of apical prostate lesions. In a previous study, the nomogram developed by Alqahtani et al.^22^ combined PSA levels and PI‐RADS to predict pathology upgrades. Jordan Nasri et al.^23^ investigated pathology upgrade within a cohort of 426 patients who underwent RP between 2014 and 2021 with an AUC of 0.87; nevertheless, their results did not separate apical tumors from non‐apical tumors, underscoring the unique contribution of our research. We believe that our nomogram can facilitate the identification of apical tumor pathology upgrades in RP specimens before surgery, thereby enhancing the strategic planning of apical dissections.

Various studies have emphasized the effect of preserving the apical complex for early recovery of continence. In general, apical dissection includes separating the prostate from the rectum posteriorly, separating neurovascular bundles laterally, dividing and ligating the dorsal venous complex, and transecting the urethra at the prostatic urethral junction. In addition, the apex is one of the most common locations of PSM and an essential site of BCR after therapy. Therefore, it is important to balance the contradiction between the large‐scale surgical resection of periprostatic tissue and the risk of PSM occurrence and dysfunction.^24^, ^25^ Generally, the classification of apical PSM as either benign or malignant is in dispute. Marcq et al.^26^ proved that the presence of focal apical positive margins did not increase the risk of BCR. Different explanations were formulated to explain why positive margins were more common at the apex, but the conclusion remains to be controversial. Reasons contributing to this considerable site‐specific PSM rate include the absence of a true anatomic prostatic capsular at the anterior side of the apex, the extreme proximity of the urethral continence mechanism requiring an adjacent surgical margin, and the dilemma in obtaining surgical exposure within a constricted space.^27^ In the present study, we observed higher PSM and BCR rates in the apical tumor pathology upgrade group than the nonupgrade group. With adequate clinical information, patients harboring clinically insignificant apical tumors at low risk of pathology upgrade could benefit from preserving the maximum length of the urethra and surrounding periurethral tissues during apical dissection, thereby offering enhanced protection of urinary continence. Conversely, patients at a high risk of pathology upgrade, as indicated by our nomogram, are recommended to undergo more aggressive management strategies.

Currently, the utilization of image‐fusion technology to minimize sampling errors through targeted biopsy represents a significant advancement. In a study, Goel et al.^28^ demonstrated that targeted biopsies are associated with significantly less pathology upgrade at surgery when compared with systematic biopsies alone. In the present study, we proved that whether a targeted biopsy was performed is a clinically significant factor in predicting apical tumor pathology. Therefore, an additional target biopsy is strongly recommended for patients with suspected apical tumor lesions based on MRI results. According to the study of Hossack et al.,^29^ different prostate biopsy approaches may have an impact on the tumor detection rate of different prostate regions and transperineal prostate biopsy performs better to detect tumors in the apical zone. In addition, transperineal prostate biopsy using a prostate mapping technique has demonstrated a higher detection rate for malignancies, coupled with a reduced incidence of postoperative infections and sepsis.^30^ A systematic review evaluating pain in 3 studies comparing transperineal versus transrectal biopsies revealed that the transperineal approach significantly increased patient pain (RR: 1.83 [1.27–2.65]).^31^ It is widely recognized that the prostate apex is a particularly sensitive area during TRUS‐guided prostate biopsies due to the dominant somatic nerve supply to the region below the dentate line.^32^ The necessity for sedation or general anesthesia arises from patient comfort considerations, given the extensive positioning required for placing a stepper and grid, in addition to the discomfort associated with each skin puncture for biopsy samples. Especially, obtaining anterior tissue of the prostate apex by TRUS‐guided prostate biopsy is a painful procedure. Kim et al.^33^ proved that the total of 14‐core prostate biopsies performed (with an extra 2 cores taken from the anterior apex) were likely to cause more severe pain at the apex compared to the conventional 10‐ to 12‐core biopsy procedures. Thus, we recommend conducting a transperineal biopsy under general anesthesia in patients with suspected apical lesions. Up to now, the optimal number of cores to be obtained during a systematic prostate biopsy, particularly in cases of negative multiparametric mpMRI and/or when complemented by a targeted fusion biopsy for the diagnosis of csPCa remains undetermined.^34^ A total of three contemporary prospective trials have explored the specific number of cores for each target during prostate biopsy: four cores in the PRECISION trial compared to three cores in the MRI‐FIRST trial, and two to four cores in the 4 M trial.^35^, ^36^, ^37^ These findings led the European Association of Urology guidelines to recommend that three to five biopsy cores be obtained for each target, while the American Association of Urology and the Society of Abdominal Radiology recommended that only two biopsy cores be obtained for each target in the consensus statement.^38^ The primary objective of targeted biopsy is to minimize the risk of overlooking PCa or undersampling lesions. Our study has proved that increasing the number of biopsies taken from apical suspicious regions of the prostate could significantly increase the accuracy of targeted biopsy. Moreover, it introduces an optimized strategy for targeting and sampling apical tumors in men, utilizing clinically significant primary mpMRI findings in conjunction with our nomogram.

Inevitably, some certain limitations in our study should be highlighted. Firstly, the study is retrospective and the bias in the process of the patient selection cannot be neglected. Nevertheless, we strictly generated the inclusion and exclusion criteria, which made the data we collected more homogeneous and truly reflective of the actual situation. Secondly, we did not perform subgroup analysis based on the low risk of PCa and the very low risk of PCa patients; this indicates the need for collecting more patient data for relevant clinical insights to refine our study in the future. Finally, our definition of an apical prostate tumor has not been validated and might be considered arbitrary; nevertheless, this definition has been applied in similar prior studies.^39^

Due to the small number of enrolled patients, we did not remove patients with high‐risk lesions in other locations when calculating BCR, resulting in biased results. In the future, our team will continue our research to provide new methods for nomograms for the diagnosis of PCa pathology upgrade. Despite these limitations, our nomogram has demonstrated its efficacy and utility as a model, effectively aiding clinicians in delivering personalized treatment strategies.

## 5 CONCLUSION

Our study introduces a comprehensive nomogram integrating clinical, radiological, and pathological data, proven to effectively predict pathology upgrades in PCa. In the apical zone, increasing the quantity of biopsy from the region of interest of the apex could significantly decrease the rate of pathology upgrade. We believe our research could provide guidance in personalizing treatment selection for patients with apical tumors, particularly those at low risk of pathology upgrade, by informing more nuanced surgical approach decisions.

## AUTHOR CONTRIBUTIONS

**Tianrui Feng:** Data curation (equal); investigation (equal); writing – review and editing (equal). **Zhen Liang:** Methodology (equal); software (equal); writing – original draft (equal). **Yu Xiao:** Conceptualization (equal); project administration (equal); resources (equal). **Boju Pan:** Data curation (equal); supervision (equal); writing – original draft (equal). **Yi Zhou:** Data curation (equal); supervision (equal); validation (equal). **Chengquan Ma:** Supervision (equal). **Zhien Zhou:** Validation (equal). **Weigang Yan:** Conceptualization (equal). **Ming Zhu:** Data curation (equal); funding acquisition (equal); visualization (equal).

## FUNDING INFORMATION

This study is funded by National High Level Hospital Clinical Research Funding (2022‐PUMCH‐A‐063 and 2022‐PUMCH‐B‐009).

## CONFLICT OF INTEREST STATEMENT

The authors declare no conflict of interest.

## Supporting information




Table S1.

Figure S1.

## Data Availability

The data that support the findings of this study are available from the corresponding author upon reasonable request.

## References

[1] Sung H , Ferlay J , Siegel RL , et al. Global Cancer Statistics 2020: GLOBOCAN estimates of incidence and mortality worldwide for 36 cancers in 185 countries. CA Cancer J Clin. 2021;71:209‐249. 33538338 10.3322/caac.21660

[2] Schütz V , Kesch C , Dieffenbacher S , et al. Multiparametric MRI and MRI/TRUS fusion guided biopsy for the diagnosis of prostate cancer. Adv Exp Med Biol. 2018;1096:87‐98. 30324349 10.1007/978-3-319-99286-0_5

[3] Prata F , Anceschi U , Cordelli E , et al. Radiomic machine‐learning analysis of multiparametric magnetic resonance imaging in the diagnosis of clinically significant prostate cancer: new combination of textural and clinical features. Curr Oncol. 2023;30:2021‐2031. 36826118 10.3390/curroncol30020157 PMC9955797

[4] Dong F , Jones JS , Stephenson AJ , Magi‐Galluzzi C , Reuther AM , Klein EA . Prostate cancer volume at biopsy predicts clinically significant upgrading. J Urol. 2008;179:896‐900. 18207180 10.1016/j.juro.2007.10.060

[5] Zhuang J , Kan Y , Wang Y , et al. Machine learning‐based prediction of pathological upgrade from combined transperineal systematic and mri‐targeted prostate biopsy to final pathology: a multicenter retrospective study. Front Oncol. 2022;12:785684. 35463339 10.3389/fonc.2022.785684 PMC9021959

[6] Cooperberg MR , Zheng Y , Faino AV , et al. Tailoring intensity of active surveillance for low‐risk prostate cancer based on individualized prediction of risk stability. JAMA Oncol. 2020;6:e203187. 32852532 10.1001/jamaoncol.2020.3187 PMC7453344

[7] Olsson H , Nordström T , Clements M , Grönberg H , Lantz AW , Eklund M . Intensity of active surveillance and transition to treatment in men with low‐risk prostate cancer. Eur Urol Oncol. 2020;3:640‐647. 31235395 10.1016/j.euo.2019.05.005

[8] Pettus JA , Weight CJ , Thompson CJ , Middleton RG , Stephenson RA . Biochemical failure in men following radical retropubic prostatectomy: impact of surgical margin status and location. J Urol. 2004;172:129‐132. 15201752 10.1097/01.ju.0000132160.68779.96

[9] Ragusa A , Brassetti A , Prata F , et al. Predictors of urinary continence recovery after laparoscopic‐assisted radical prostatectomy: is surgical urethral length the only key factor? Life (Basel). 2023;13:1550. 37511925 10.3390/life13071550 PMC10381846

[10] Ishii J , Ohori M , Scardino P , Tsuboi T , Slawin K , Wheeler T . Significance of the craniocaudal distribution of cancer in radical prostatectomy specimens. Int J Urol. 2007;14:817‐821. 17760748 10.1111/j.1442-2042.2007.01836.x

[11] van Dessel LF , Reuvers SHM , Bangma CH , Aluwini S . Salvage radiotherapy after radical prostatectomy: long‐term results of urinary incontinence, toxicity and treatment outcomes. Clin Transl Radiat Oncol. 2018;11:26‐32. 30014044 10.1016/j.ctro.2018.05.001 PMC6019864

[12] Gao X , Wang KB , Pu XY , Zhou XF , Qiu JG . Modified apical dissection of the prostate improves early continence in laparoscopic radical prostatectomy: technique and initial results. J Cancer Res Clin Oncol. 2010;136:511‐516. 19774396 10.1007/s00432-009-0683-4 PMC11827937

[13] Prata F , Anceschi U , Taffon C , et al. Real‐time urethral and ureteral assessment during radical cystectomy using ex‐vivo optical imaging: a novel technique for the evaluation of fresh unfixed surgical margins. Curr Oncol. 2023;30:3421‐3431. 36975472 10.3390/curroncol30030259 PMC10047830

[14] Iasonos A , Schrag D , Raj GV , Panageas KS . How to build and interpret a nomogram for cancer prognosis. J Clin Oncol. 2008;26:1364‐1370. 18323559 10.1200/JCO.2007.12.9791

[15] Mohler JL , Antonarakis ES , Armstrong AJ , et al. Prostate cancer, version 2.2019, NCCN clinical practice guidelines in oncology. J Natl Compr Cancer Netw. 2019;17:479‐505. 10.6004/jnccn.2019.0023 31085757

[16] Weinreb JC , Barentsz JO , Choyke PL , et al. PI‐RADS prostate imaging—reporting and data system: 2015, version 2. Eur Urol. 2016;69:16‐40. 26427566 10.1016/j.eururo.2015.08.052 PMC6467207

[17] Landis JR , Koch GG . The measurement of observer agreement for categorical data. Biometrics. 1977;33:159‐174. 843571

[18] Diamand R , Peltier A , Roche JB , et al. Optimizing multiparametric magnetic resonance imaging‐targeted biopsy and prostate cancer grading accuracy. World J Urol. 2023;41:77‐84. 36509932 10.1007/s00345-022-04244-4

[19] Liao X , Qiao P , Tan Z , Shi H , Xing N . "Total reconstruction" of the urethrovesical anastomosis contributes to early urinary continence in laparoscopic radical prostatectomy. Int Braz J Urol. 2016;42:215‐222. 27256174 10.1590/S1677-5538.IBJU.2014.0666 PMC4871380

[20] Nattino G , Finazzi S , Bertolini G . A new test and graphical tool to assess the goodness of fit of logistic regression models. Stat Med. 2016;35:709‐720. 26439593 10.1002/sim.6744

[21] Calleris G , Marra G , Benfant N , et al. Salvage radical prostatectomy for recurrent prostate cancer following first‐line nonsurgical treatment: validation of the European Association of Urology criteria in a large, multicenter, contemporary cohort. Eur Urol Focus. 2023;9:645‐649. 36682962 10.1016/j.euf.2023.01.006

[22] Alqahtani S , Wei C , Zhang Y , et al. Prediction of prostate cancer Gleason score upgrading from biopsy to radical prostatectomy using pre‐biopsy multiparametric MRI PIRADS scoring system. Sci Rep. 2020;10:7722. 32382097 10.1038/s41598-020-64693-y PMC7205887

[23] Nasri J , Barthe F , Parekh S , et al. Nomogram predicting adverse pathology outcome on radical prostatectomy in low‐risk prostate cancer men. Urology. 2022;166:189‐195. 35263642 10.1016/j.urology.2022.02.019

[24] Cumarasamy S , Martini A , Falagario UG , et al. Development of a model to predict prostate cancer at the apex (PCAP model) in patients undergoing robot‐assisted radical prostatectomy. World J Urol. 2020;38:813‐819. 31435731 10.1007/s00345-019-02905-5

[25] Wright JL , Ellis WJ . Improved prostate cancer detection with anterior apical prostate biopsies. Urol Oncol. 2006;24:492‐495. 17138129 10.1016/j.urolonc.2006.03.003

[26] Marcq G , Michelet A , Hannink G , et al. Risk of biochemical recurrence based on extent and location of positive surgical margins after robot‐assisted laparoscopic radical prostatectomy. BMC Cancer. 2018;18:1291. 30587172 10.1186/s12885-018-5229-1 PMC6307117

[27] Kübler HR , Szukala SA , Madden JF , et al. Apical soft tissue biopsies predict biochemical failure in radical perineal prostatectomy patients with apical cancer involvement. Prostate Cancer Prostatic Dis. 2007;10:72‐76. 17179978 10.1038/sj.pcan.4500926

[28] Goel S , Shoag JE , Gross MD , et al. Concordance between biopsy and radical prostatectomy pathology in the era of targeted biopsy: a systematic review and meta‐analysis. Eur Urol Oncol. 2020;3:10‐20. 31492650 10.1016/j.euo.2019.08.001

[29] Hossack T , Patel MI , Huo A , et al. Location and pathological characteristics of cancers in radical prostatectomy specimens identified by transperineal biopsy compared to transrectal biopsy. J Urol. 2012;188:781‐785. 22819419 10.1016/j.juro.2012.05.006

[30] Skouteris VM , Crawford ED , Mouraviev V , et al. Transrectal ultrasound‐guided versus transperineal mapping prostate biopsy: complication comparison. Rev Urol. 2018;20:19‐25. 29942197 10.3909/riu0785 PMC6003299

[31] Xiang J , Yan H , Li J , Wang X , Chen H , Zheng X . Transperineal versus transrectal prostate biopsy in the diagnosis of prostate cancer: a systematic review and meta‐analysis. World J Surg Oncol. 2019;17:31. 30760274 10.1186/s12957-019-1573-0 PMC6375152

[32] Nazir B . Pain during transrectal ultrasound‐guided prostate biopsy and the role of periprostatic nerve block: what radiologists should know. Korean J Radiol. 2014;15:543‐553. 25246816 10.3348/kjr.2014.15.5.543 PMC4170156

[33] Kim SJ , Lee J , An DH , et al. A randomized controlled comparison between periprostatic nerve block and pelvic plexus block at the base and apex of 14‐core prostate biopsies. World J Urol. 2019;37:2663‐2669. 30864006 10.1007/s00345-019-02722-w

[34] Pepe P , Pennisi M , Fraggetta F . How many cores should be obtained during saturation biopsy in the era of multiparametric magnetic resonance? Experience in 875 patients submitted to repeat prostate biopsy. Urology. 2020;137:133‐137. 31758981 10.1016/j.urology.2019.11.016

[35] Kasivisvanathan V , Rannikko AS , Borghi M , et al. MRI‐targeted or standard biopsy for prostate‐cancer diagnosis. N Engl J Med. 2018;378:1767‐1777. 29552975 10.1056/NEJMoa1801993 PMC9084630

[36] Rouvière O , Puech P , Renard‐Penna R , et al. Use of prostate systematic and targeted biopsy on the basis of multiparametric MRI in biopsy‐naive patients (MRI‐FIRST): a prospective, multicentre, paired diagnostic study. Lancet Oncol. 2019;20:100‐109. 30470502 10.1016/S1470-2045(18)30569-2

[37] van der Leest M , Cornel E , Israël B , et al. Head‐to‐head comparison of transrectal ultrasound‐guided prostate biopsy versus multiparametric prostate resonance imaging with subsequent magnetic resonance‐guided biopsy in biopsy‐naïve men with elevated prostate‐specific antigen: a large prospective multicenter clinical study. Eur Urol. 2019;75:570‐578. 30477981 10.1016/j.eururo.2018.11.023

[38] Rosenkrantz SAB , Verma S , Choyke P . American Urological Association (AUA) Society of Abdominal Radiology (Sar) Joint Consensus Statement: prostate MRI and MRI‐Targeted biopsy in patients with prior negative biopsy. 2016.

[39] Kenigsberg AP , Tamada T , Rosenkrantz AB , et al. Multiparametric magnetic resonance imaging identifies significant apical prostate cancers. BJU Int. 2018;121:239‐243. 28805295 10.1111/bju.13987

