# Supplementary material for: Can a nomogram predict apical prostate cancer pathology upgrade from fusion biopsy to final pathology? A multicenter study
Source: Cancer Med. 2024 Jun 7;13(11):e7341. doi: 10.1002/cam4.7341 (PMC11157165; doi:10.1002/cam4.7341)
Supplement: Supplementary file 1 — Table S1. [file CAM4-13-e7341-s002.doc]

**1 Supplementary Table 1. Prostate multiparametric magnetic resonance imaging protocol**

| Parameters | T2WI | DWI |
| --- | --- | --- |
| Sequence | FRFSE | SE-EPI |
| TR/TE (ms) | 4137/86 | 4200/90 |
| Flip angle (degree) | 110 | 90 |
| Echo train length | 32 | 1 |
| Field of view (mm × mm) | 270 × 270 | 360 × 360 |
| Matrix size | 288 × 192 | 128 × 96 |
| Thickness (mm) | 3.0 | 3.0 |
| Other |  | b values = 0, 100, 150, 200, 500, 800, 1000, 1500, 2000 mm^2^/sec |
