# Supplementary figures and images for: Can a nomogram predict apical prostate cancer pathology upgrade from fusion biopsy to final pathology? A multicenter study
Source: Cancer Med. 2024 Jun 7;13(11):e7341. doi: 10.1002/cam4.7341 (PMC11157165; doi:10.1002/cam4.7341)

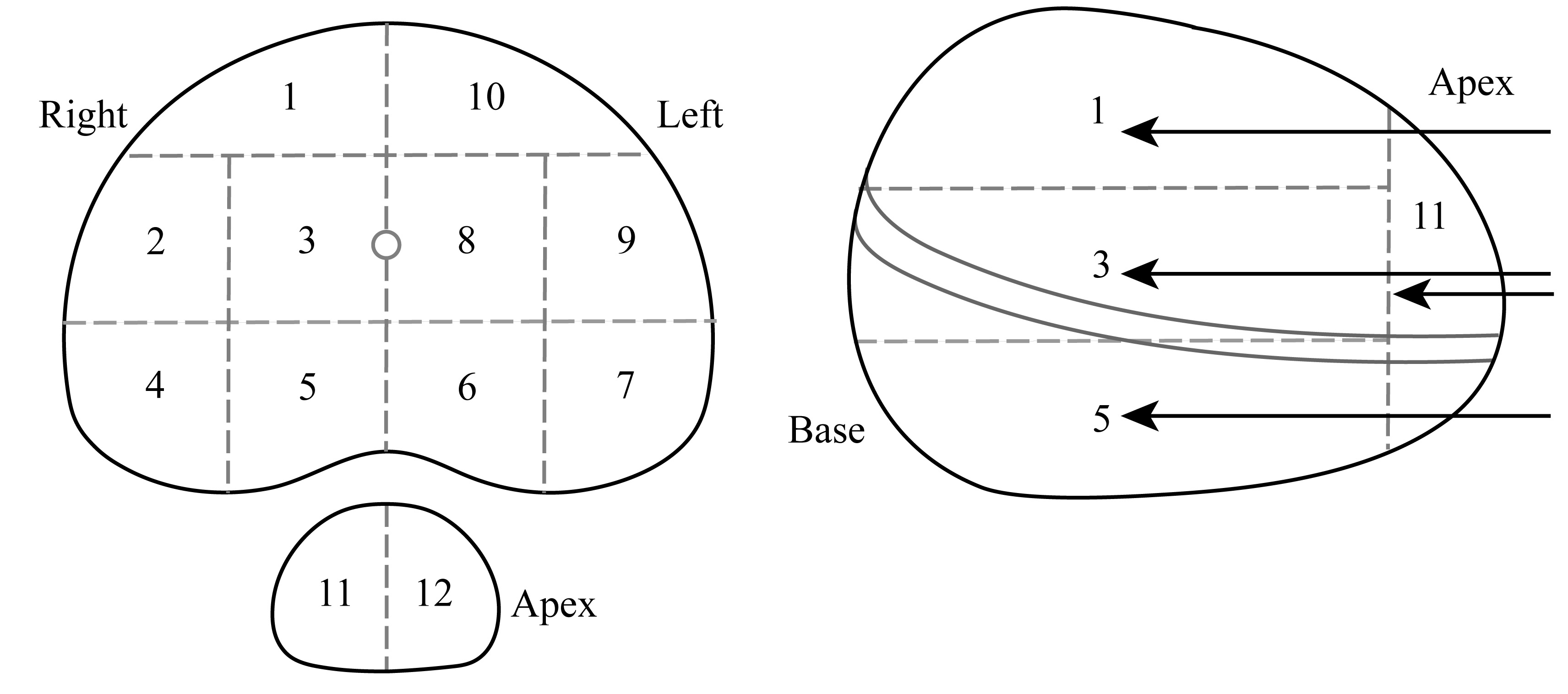

Supplement: Supplementary file 2 — Figure S1. [file CAM4-13-e7341-s001.jpg]
